# Can testing clinical significance reduce false positive rates in randomized controlled trials? A snap review

**DOI:** 10.1186/s13104-017-3117-4

**Published:** 2017-12-28

**Authors:** Theophile Bigirumurame, Adetayo S. Kasim

**Affiliations:** Durham University, University boulevard, Stockton-on-Tees, TS17 6BH UK

**Keywords:** Null hypothesis testing, Minimum clinically important difference, Sample size calculation, False discovery

## Abstract

**Objective:**

The use of minimum clinically important difference in the hypothesis formulation for superiority trials is similar in principle to the concept of non-inferiority or equivalence trial. However, most clinical trials are analysed testing zero clinical difference. Since the minimum clinically important difference is pre-defined for power calculation, it is important to incorporate it in both the hypothesis testing and the interpretation of findings from clinical trials.

**Results:**

We reviewed a set of 50 publications (25 with binary outcome, and 25 with survival time outcome). 20% of the 50 published trials that were statistically significant, were also clinically significant based on the minimum clinically important risk differences (or hazard ratio) used for their power calculations. This snap review seems to suggest that most published trials with statistically significant results were less likely to be clinically significant, which may partly explain the high false positive findings associated with findings from superiority trials. Furthermore, none of the reviewed publications explicitly used minimum clinically important difference in the interpretation of their findings. However, a systematic review is needed to critically appraise the impact of the current practice on false positive rate in published trials with significant findings.

## Introduction

A lot has been said over the years about the limitation of null hypothesis significance testing, particularly with respect to false positive rates among the published findings [1]. What is also clear, is that the main problem is how the null hypothesis significance testing is used rather than the statistical approach itself [2]. One cause for concern is a mismatch between hypothesis formulation for power calculation and the hypothesis assumed for analysing trial data. Typically, sample size calculation for randomised controlled trials is based on a minimum clinically important difference (MCID) that should support both statistical significance and clinical findings from the trial. The objective of this snap review is to investigate how often the pre-specified minimum clinically important difference is used in the interpretation of findings from a clinical trial. We also aim to show that most published trials with statistical significant results are not necessarily clinically significant, which we believe may partly explain the high false positive rate associated with findings from superiority trials.

## Main text

Let $\delta$ be the MCID, then a 2-sided hypothesis for power calculation taking into account both statistical and clinical significance can be formulated as:

$$\begin{aligned} \begin{array}{l} H_0:-\delta \le \mu _2-\mu _1 \le \delta , \\ H_1:\mu _2-\mu _1 \le -\delta \quad or \quad \mu _2-\mu _1 \ge \delta . \end{array} \end{aligned}$$

where: $\mu _1$ and $\mu _2$ are the expected means for the control (or placebo) and the active groups, respectively (if we consider a normally distributed outcome).

The use of MCID in the hypothesis formulation for superiority trials is similar in principle to the concept of non-inferiority or equivalence trials. However most clinical trials are analysed assuming zero clinical difference based on the hypothesis:

$$\begin{aligned} \begin{array}{l} H_0:\mu _2-\mu _1 =0, \\ H_1:\mu _2-\mu _1 \ne 0. \end{array} \end{aligned}$$

**Fig. 1:**
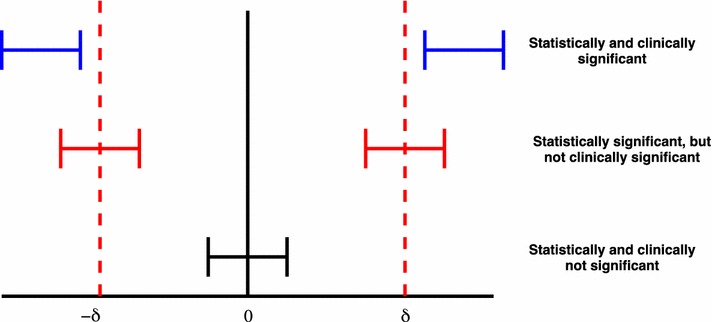
Statistical and clinical significance evaluated using confidence intervals

The clinical relevance of the findings is left to the qualitative judgement of clinicians and researchers. Since MCID has been pre-defined for power calculation, it is natural to incorporate this value in testing and interpreting results from clinical trials. A framework that was proposed by [3] and [4] is using confidence interval as illustrated in Fig. 1.

We performed our snap review by searching all published parallel group randomised trials using the search terms “randomised controlled trials”, “randomized controlled trials”, “phase 3”, “superiority trials” for articles published in *New England Journal of Medicine* and *The Lancet* between 2004 and March 2017. We have only considered the two journals in our snap review because of the high quality of their publications, likelihood to report relevant information on MCID and the limited resources on our side to do this snap review. We acknowledge that restricting our review to only the two journals may imply that our conclusion may be biased and may not be broadly applicable to all published trials. However, we believe the objective of this snap review remains valid and timely to warrant a future systematic review of this topic. We screened abstracts and result sections of the papers to identify those published with significant results (P values < 0.05). We excluded articles which did not report minimum clinically important difference used for sample size calculation. For dichotomous outcomes, we noted that different statistics were used to estimate treatment effects (odds ratio, relative risk, relative risk reduction, risk difference). We expressed MCID and treatment effects as risk difference (proportion in the active treatment group minus the proportion in the control group). Confidence intervals were computed using the normal approximation. For time to event outcomes, we only selected publications in which the MCID and treatment effects were expressed as hazard ratio (HR). Since there were very few publications with continuous outcomes, we focused only on dichotomous and survival outcomes.

**Fig. 2:**
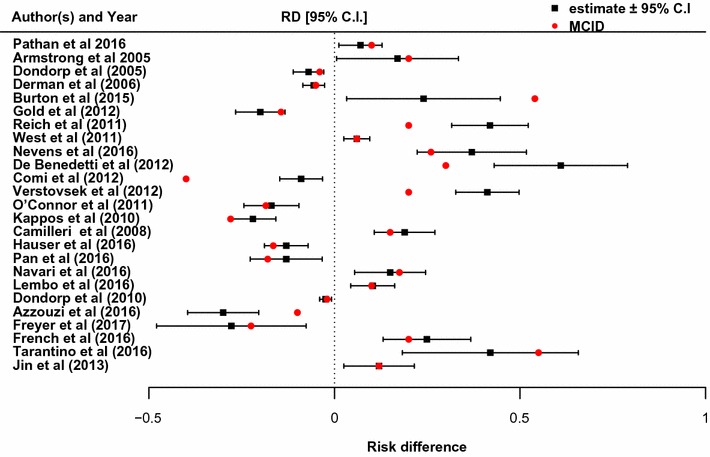
Estimated risk differences ± 95% C.I and corresponding MCID

Selected publications were then grouped into two categories based on the primary endpoint types (survival or binary endpoint). Figure 2 presents selected publications for binary endpoint group.

**Fig. 3:**
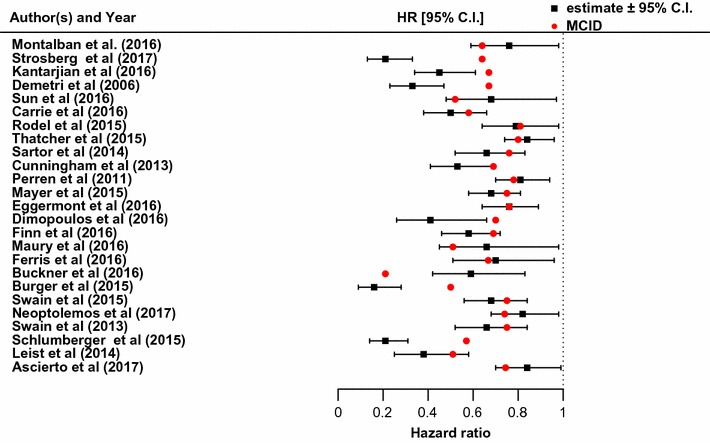
Estimated hazard ratio ± 95% C.I and corresponding MCID

The vertical dashed line shows the zero difference. As expected, the estimated risk differences ± 95% confidence interval (C.I.) lie on either side of the zero line depending on whether the goal of the trial is to establish positive or negative impact of the interventions (improvement in response rate or reduction in risk rate). In Fig. 2, 4 of the 25 (16%) publications reported risk differences (RD) which were both statistically and clinically significant with estimated RD and confidence intervals bounds greater than their pre-specified MCID (in absolute value). 2 of the 25 (8%) publications reported RD which were statistically significant but not clinically significant with estimated RD and confidence intervals bounds smaller than their pre-specified MCID (in absolute value). The remaining trials (76%) reported statistically significant but not necessarily clinically significant RD since their pre-specified MCID were within the bounds of their estimated confidence intervals. For the survival endpoint, selected publications are shown in Fig. 3.

6 of the 25 (24%) publications reported HR which were both statistically and clinically significant. 1 of the 25 (4%) publications reported HR which was statistically significant but not clinically significant. The remaining 72% of publications reported HR which were statistically significant but not necessarily clinically significant. Combining results from the two groups, it can be shown that only 10 of the 50 (20%) publications reported estimates which were both statistically and clinically significant. 6% of the published results were statistically significant but not clinically significant. The remaining 74% of publications were statistically significant but not necessary clinically significant. Comparable results were reported by [5] in their investigation of publicly funded trials. Furthermore, none of the reviewed publications explicitly used MCID in the interpretation of their findings.

## Limitations

Our findings support the need to improve the way hypothesis testing is conducted for superiority trials. In most superiority trials, the MCID is only used in the sample size calculation. This approach should be improved by taking into account all the information used in sample size calculation during the analysis and interpretation of the trial data. To improve reliability of published results, investigators should do more to report all the necessary information used for sample size calculation.

This snap review seems to suggest that most published trials with statistically significant results were less likely to be clinically significant, which may partly explain the high false positive findings associated with findings from superiority trials. However, a systematic review is needed to critically appraise the impact of the current practice on false positive rates in published trials with significant findings. In addition to a systematic a review, our future research will investigate the degree of association between clinical significance and false positive findings by assessing whether findings that are both statistically and clinically significant have smaller false positive rate. We would also like to investigate whether Bayesian local false discovery rate can predict false positive findings in published trials. This snap review assumed that the minimum clinically important difference has been correctly specified, guidance on how to best determine the most plausible minimum clinically important difference for a trial is provided by DELTA^2^ initiative [6].

The review of publications from only two journals is a major limitation because our conclusion may not be broadly applicable to all published journals. We chose these two journals because of the high quality of their publications and the likelihood to report the relevant information on sample size calculation, particularly the assumed MCID. Another limitation is that the snap review and our conclusions assumed that the sample size calculation have correctly been determined and that the value specified as MCID is correct. However, determining appropriate MCID is not straight forward and DELTA^2^ proposed different ways to determine MCID. Lastly, this snap review did not follow a rigorous systematic review approach and the criteria used to select the publications may suffer from selection bias. In spite of these limitations, we believe that this snap review is informative given the ongoing concerns about significance hypothesis testing and lack of reproducibility of published findings from clinical trials.

## Abbreviations

MCID: minimum clinically important difference
HR: hazard ratio
RD: risk difference
C.I: confidence interval

## References

[1] Ioannidis JP (2005). Why most published research findings are false. PLos med.

[2] Robinson DH (2001). On the past and future of null hypothesis significance testing. ETS Res Rep Series.

[3] Shafiq N, Malhotra S (2015). Superiority trials: raising the bar of null hypothesis statistical testing. Evid Based Med.

[4] Ranstam J (2012). Why the P-value culture is bad and confidence intervals a better alternative. Osteoarthr Cartil.

[5] Sully BG, Julious SA, Nicholl J (2014). An investigation of the impact of futility analysis in publicly funded trials. Trials.

[6] Cook JA, Julious SA, Sones W, Rothwell JC, Ramsay CR, Hampson LV (2017). Choosing the target difference (‘effect size’) for a randomised controlled trial-DELTA 2 guidance protocol. Trials.

